# Early retirement intentions of Korean wage earners: the influence of job demand-control-support latent profiles

**DOI:** 10.1186/s12889-025-23158-5

**Published:** 2025-06-06

**Authors:** Ara Jo, Hye-Sun Jung

**Affiliations:** Department of Preventive Medicine, College of Medicine, The Catholic University of Korea, 222 Banpo- daero, Seocho-gu, Seoul, 06591 Republic of Korea

**Keywords:** Early retirement, Korea, Wage earners, Job control, Job demand, Job support, Latent profile analysis

## Abstract

**Background:**

The global aging of the population is a serious issue, and with working life expectancy increasing, it is crucial to consider measures to delay retirement. Since retirement intention is a key factor in determining the timing of retirement, understanding the psychological state of workers as a determinant of retirement intention is necessary. The purpose of this study is twofold: (1) to identify latent profile types based on job demands, control, and support among Korean wage earners using a person-centered approach; and (2) to examine the association between these latent profiles and early retirement intentions.

**Methods:**

We analyzed data from 31,587 wage-earning participants aged 19 to 59 using the sixth Korean Working Conditions Survey (KWCS), conducted between 2020 and 2021. The sample included 57.06% men and 42.94% women. Latent Profile Analysis (LPA), a person-centered statistical method used to identify unobserved subgroups within a population, was employed to classify participants into five job characteristic profiles based on the job demand-control-support (JDCS) model. Job demands were measured across physical, quantitative, emotional, and social aspects; job control was assessed by items related to autonomy in task execution; and job support included perceived support from supervisors and coworkers. Early retirement intention, the outcome variable, was measured by asking participants the age until which they intended to work. Multivariate logistic regression analysis was conducted to examine the association between the identified job profiles and early retirement intentions, adjusting for relevant sociodemographic and occupational covariates.

**Results:**

Five latent profile types were identified based on levels of job demands, control, and support using LPA. These profiles were labeled according to the Job Demand-Control-Support (JDCS) model and named as follows: Low Strain Collective (5.52%), Active Collective (27.99%), Passive Collective (28.92%), High Strain Collective (32.56%), and Low Strain Isolated (5.01%). The names reflect the distinct combinations of job demand, control, and support characteristics within each group. Multivariate logistic regression analysis showed that, compared to the Low Strain Collective, the Active Collective (OR = 1.65, 95% CI = 1.10–2.48), Passive Collective (OR = 1.72, 95% CI = 1.15–2.60), and High Strain Collective (OR = 1.66, 95% CI = 1.10–2.49) groups had significantly higher early retirement intentions. Additionally, gender, age group, education level, household income contribution, occupation type, employment type, and presenteeism were significantly associated with early retirement intentions.

**Conclusion:**

Our findings suggest that to reduce early retirement intentions, employees should be given jobs that consider their personal and work characteristics, and they should have an appropriate level of job control. Moreover, creating a supportive atmosphere from supervisors and coworkers is essential.

## Background

The aging population is a serious problem both domestically and internationally, prompting several governments to extend the retirement age as a solution to reduce the socioeconomic burden [1, 2]. People aged 65 and older made up 16.5% of South Korea’s population this year, highlighting concerns that the fast-aging demographic transition could pose a drag on the country’s economy [3]. Extending working life may have beneficial effects on the physical and mental health and quality of life of older workers [4, 5]. The legal retirement age implemented in Korea in 2016 is 60 years old, while the starting age for receiving a national pension is 65 years old, creating a mismatch between the retirement age system and the age for receiving old-age pension benefits [6]. Nevertheless, in Korea, there are difficulties in maintaining employment until the legal retirement age, and the probability of retiring before that age is increasing [1]. It has been found that early retirement is influenced not only by the employee’s health but also by factors such as job demands and job control [7]. Compounding this issue is the mismatch between Korea’s legal retirement age (60 years) and the national pension eligibility age (65 years), which has led to increased income gaps for retirees. Unlike other countries with more flexible retirement systems or early workforce exit options, Korea faces challenges due to stringent employment practices and limited old-age income security. Early retirement exacerbates socioeconomic issues by reducing the labor force and increasing dependence on public welfare systems.

RA Karasek [8]RA Karasek [8] developed a job demand-control (JDC) model to explain job characteristics and the psychological state of workers. The model proposes that biologically adverse strain arises when the psychological demands of a job exceed the resources available for task control. The combination of high demands and low control serves as a major factor in causing job strain. In addition, the Job Demand-Control-Support (JDCS) model was introduced by incorporating the concept of “social support” into the existing JDC model [9]. The key aspect of the JDCS model is that the combination of job demands, job control, and social support increases the risk of higher strain or reduced well-being, beyond the individual effects of each factor [9]. A human-centered approach was adopted to identify subgroups of individuals with different patterns, specifically occupational type profiles. By adopting a person-centered approach using this model, it is possible to identify subgroups with different patterns, specifically occupational type profiles. Johnson and Hall categorized work-related social support into isolated and collective conditions, thereby reframing the concept of job strain [10].

For identifying issues such as job factors, psychosocial factors, and health that affect retirement intentions, European countries have conducted studies on various occupations and age groups [11–15].

A 2018 systematic review found evidence that high job control and job satisfaction influence later retirement intentions and actual retirement [16]. A growing body of early retirement intentions research suggests that for workers aged 50 and older and just before the normal retirement age, heavy job demands, such as strenuous physical labor, job stress [17], recognition and support at work [18, 19], health status [12, 13, 19], job satisfaction [14], and effort-reward imbalance [12, 13], are significant factors. Additionally, studies have identified early retirement intentions in specific occupational groups. Workplace support from supervisors and coworkers influenced early retirement intentions among police officers [20]. For construction workers, strenuous physical labor that can cause pain was a major factor [21]. For healthcare workers, high job demands [22], job satisfaction [23–25], organizational role [26, 27], and burnout [28] were significant factors.

However, to the best of our knowledge, no studies have been conducted using Latent Profile Analysis (LPA) to identify subgroups with similar job profiles (JDCS) and how each group is differently associated with early retirement intentions. Moreover, existing studies have analyzed early retirement intentions using job demands, job control, and social support factors individually [29, 30]. However, these studies often lacked a comprehensive framework and failed to account for latent subgroups with differing workplace experiences. This study addresses these limitations by employing the JDCS model and LPA to explore multidimensional job profiles, providing a more nuanced understanding of early retirement intentions and offering actionable insights for workforce retention policies.

In contrast, in Korea, only studies on specific occupations have been published [30]. Traditional studies that assess each of the variables of job demands, control, and support separately in relation to early retirement intentions may fail to uncover individual worker characteristics [31]. On the other hand, LPA is a statistical method that uses a person-centered approach to identify potential groups within a measurable continuous variable. LPA has the advantage of dividing the population into different subgroups to identify characteristics [32]. Therefore, LPA can be used to identify subgroups of Korean wage earners with similar levels of job demands, control, and support, and to determine the extent to which each group influences early retirement intentions. This study aims to (1) investigate the job characteristics profile of Korean wage earners using LPA based on the JDCS model and (2) identify the association between JDCS profiles and early retirement intentions. Accordingly, the research questions of this study are (1) What are the latent job characteristic profiles of Korean wage earners based on the JDCS model? (2) How are these profiles associated with early retirement intentions? (3) How do these findings inform workforce retention policies in an aging society?

This study is original in its application of the JDCS model using LPA to explore early retirement intentions among Korean wage earners, a context where such comprehensive analyses are rare. Unlike prior research focusing on individual job factors or specific occupational groups, this study identifies latent job profiles by combining job demands, control, and support. This person-centered approach allows for nuanced insights into how different combinations of workplace characteristics influence retirement intentions. The study contributes to the literature by addressing gaps in understanding the interplay of psychosocial factors and workforce retention. It also informs policymakers and organizations on designing tailored interventions to manage job demands and enhance workplace support, ultimately reducing premature workforce exits.

## Theoretical background

The aging population and increasing retirement intentions present critical challenges to workforce sustainability and socioeconomic stability globally, particularly in Korea. Numerous studies have explored the factors influencing early retirement intentions, identifying key variables such as job demands, job control, social support, and individual health status [16, 29]. However, the interplay of these factors and their combined effects on early retirement intentions remains underexplored.

The JDCS model, originally developed as the Job Demand-Control (JDC) model [8], provides a comprehensive framework for understanding workplace stress. The JDC model posits that job strain arises when high job demands exceed the resources provided by job control, defined as the ability to influence tasks, decisions, and the broader work environment. JV Johnson and EM Hall [10]JV Johnson and EM Hall [10] later expanded this model to include social support from coworkers and supervisors, creating the JDCS model. Social support acts as a buffer against stress, mitigating the negative impact of job strain and enhancing worker well-being. This model is particularly useful in analyzing retirement intentions, as workplace demands, control, and support directly influence job satisfaction, health, and long-term employment decisions [18, 33].

Job demands refer to the physical, psychological, and social requirements of a job, such as strenuous physical labor, tight deadlines, or emotional labor. High job demands have been linked to increased job stress, health deterioration, and early retirement intentions. For example, studies have found that heavy physical labor among construction workers contributes to early retirement intentions due to chronic pain and physical fatigue [21]. Similarly, healthcare workers experience high job demands through emotionally taxing tasks, which, combined with burnout and reduced job satisfaction, lead to premature retirement [22, 28].

Job control encompasses an employee’s autonomy in decision-making, task scheduling, and work-related choices. Low job control has been associated with higher job strain and a stronger preference for early retirement [34]. Conversely, high job control can promote job satisfaction and reduce stress, delaying retirement intentions [17].

Social support from supervisors and coworkers significantly impacts retirement intentions by enhancing job satisfaction and reducing stress. Workplace support fosters a sense of belonging, motivation, and confidence, which counteracts the adverse effects of job demands [18, 20]. For police officers, support from supervisors and coworkers has been shown to lower early retirement intentions by improving emotional resilience and stress management [20]. In contrast, the absence of workplace support exacerbates stress and dissatisfaction, increasing the likelihood of premature retirement [15].

Research has also highlighted the combined effects of these factors. For instance, recognition and support at work reduce early retirement intentions by fostering a sense of value and fairness [19]. However, traditional studies assessing these variables in isolation often fail to capture the nuanced interplay between demands, control, and support, which collectively shape workers’ retirement decisions. LPA, a person-centered statistical method, addresses this gap by identifying subgroups with similar job profiles and analyzing their unique influences on early retirement intentions [32].

Previous studies in European countries have examined retirement intentions across various occupations and age groups, providing insights into how different workplace factors interact to influence retirement [16]. However, in Korea, research has primarily focused on specific occupations, overlooking broader trends and subgroup-specific differences [30]. This study aims to fill this gap by applying the JDCS model and LPA to identify latent job profiles among Korean wage earners and analyze their association with early retirement intentions.

## Methods

### Data source and sample

This study involved a secondary analysis of data from the sixth wave of the Korean Working Conditions Survey (KWCS), conducted by the Korean Occupational Safety and Health Agency in 2020(https://oshri.kosha.or.kr/oshri/researchField/downWorkingEnvironmentSurvey.do). The KWCS aims to gather information on the sociodemographic characteristics, working conditions, and health of Korean workers. The survey targets all workers aged 15 and older residing in South Korea. The KWCS is similar in content and structure to the European Working Conditions Survey or the British Labour Force Survey. The KWCS utilizes a multi-staged systemic sampling method, with the enumeration district serving as a stratifying variable. The sixth wave of the KWCS included 50,538 survey participants, with data collected from October 2020 to April 2021 through one-on-one, face-to-face interviews conducted by professional interviewers from the OSHRI. For this study, the initial sample consisted of 50,538 respondents from the sixth Korean Working Conditions Survey. After applying the inclusion criteria, the final analytical sample comprised 31,587 wage earners aged 19 to 59 employed at establishments with two or more workers.

### Measures and instruments

Several instruments and items were used to examine the association between job demands, control, and support profiles and early retirement intentions of Korean wage earners.

#### Job demand-control-support

The job demands, control, and support model (JDCS model) for identifying job characteristics forms the basis of this study and is consistent with previous research on the model [31, 35] and prior research on each of its sub-variables [31, 35–37]. The JDCS model, an extension of Karasek’s JDC model, examines how job demands, control, and social support collectively influence workplace stress and well-being [8, 10]. High demands combined with low control and minimal support can lead to job strain, affecting job satisfaction, health, and retirement intentions. This model is widely used to analyze workforce dynamics and retention strategies.

Job demands consisted of 27 items in four parts: 14 physical demands, 2 quantitative demands, 4 emotional demands, and 7 social demands. Job control consisted of 3 items, and job support consisted of 7 items. Physical demands measured items related to three types of work-related hazards: ambient environment (exposure to vibration, noise, and high or low temperatures), biochemical hazards (exposure to fumes, dust, powder, chemicals, and handling infectious materials), and posture-related hazards (uncomfortable posture, lifting or carrying heavy objects, repetitive hand movements, etc.). The survey questions covered exposure to factors such as vibrations from hand tools or machinery, handling or skin contact with chemical products or substances, and working in physically straining or pain-inducing postures. Quantitative demands included questions about working at a very fast pace or to strict deadlines, while emotional demands included questions about dealing with angry customers or patients, being put in emotionally disturbing situations, and hiding emotions. Questions on quantitative demands addressed the frequency of working at very high speeds or under strict deadlines. Emotional demands included questions about how often participants directly interacted with non-colleagues such as customers, passengers, students, or patients, encountered emotionally unsettling situations, or suppressed their emotions while working. Social demands referred to exposure to adverse social behavioral conditions, such as humiliating, abusive, or violent behavior, and included questions about being insulted, victimized by sexual harassment, and experiencing workplace bullying. Questions on social demands focused on experiences of verbal abuse, unwanted sexual attention, physical violence, or bullying/harassment. Each variable allowed responses such as “exposed throughout the entire workday,” “most of the time,” “half of the time,” “rarely,” or “not at all,” with each answer assigned a numerical value from 1 to 2. The sum of these scores was used as the aggregated score for analysis. The Cronbach’s α for each of the four sub-variables of job demands was 0.89 for physical demands, 0.86 for quantitative demands, 0.65 for emotional demands, and 0.70 for social demands. The job control indicator included items related to work flexibility and decision-making authority (e.g., taking breaks when desired, being able to influence important decisions about work) and had a Cronbach’s α of 0.64. The job support measure was divided into supervisor and coworker support items, which included questions such as whether my supervisor or coworkers help and support me, and whether my supervisor treats me with respect or helps me get things done. The job support scale had a Cronbach’s α of 0.89. The authors scored the sum of each job demands-control-support score. For each variable, respondents could choose from options such as “exposed throughout the entire workday,” “most of the time,” “half of the time,” “rarely,” or “not at all.” Each response was assigned a numerical value ranging from 1 to 2, and the total score was calculated by summing these values.

#### Early retirement intentions

Early retirement intentions have been conceptualized in prior research as the willingness to work until the official retirement age or the consideration of retirement before the normal retirement or legal pension eligibility age [38–40]. These studies often assessed early retirement intentions by asking whether individuals planned to retire before the nationally recognized retirement age. In the Korean context, where the legal retirement age is 60 and the national pension eligibility age is 65, we measured early retirement intentions using participants’ numerical responses to the question of how old they intended to work, allowing for an analysis aligned with Korea’s unique retirement system. Participants could fill in the age at which they wanted to continue working or answer “as long as possible.”

This research separated the responses into two groups: those who answered “59 years old or younger” and those who answered “60 years old or older” or “as long as possible.” This is based on the legal retirement age of 60, as per the 2016 amendment to the Korean law [1], and can be interpreted to mean that respondents who want to work until age 59 want to stop working earlier than the legal retirement age. Therefore, we used the answers to the question of how old Korean wage earners want to work to divide them into two groups: those with early retirement intentions and those without.

#### Covariates

The covariates included in this study were selected based on previous literature demonstrating their association with early retirement intentions. Socio-demographic characteristics—such as gender, age, income level, education level, and household income contribution—have been shown to significantly influence retirement decisions [41, 42]. Work-related characteristics, including occupation, employment type, tenure, and shift work, affect job security and satisfaction, thereby shaping retirement intentions. Family conflict was included as a covariate given its documented effect on elevating stress and work-life imbalance, which may increase the likelihood of early retirement [43]. Health problems were also considered, as poor physical or mental health is a strong predictor of early labor market exit, particularly in high-demand occupations such as emergency services [44]. Lastly, presenteeism—defined as attending work while ill—was included due to its known impact on exacerbating physical and emotional exhaustion, which can accelerate retirement decisions [45].

### Data availability and ethics statement

Data from the 6th KWCS are publicly available and can be found at https://www.kosha.or.kr. Since this study involved the analysis of secondary data, it was exempt from review. Exemption from review was obtained from the Institutional Review Board of the Catholic University (MC24ZISI0066), which waived the informed consent requirement.

### Data analysis

Descriptive statistics were used to summarize the sociodemographic and occupational characteristics of the participants and the main study variables. LPA was conducted based on the JDCS model to classify participants into distinct job characteristic profiles. Model selection was guided by fit indices including the Akaike Information Criterion (AIC), Bayesian Information Criterion (BIC), Sample-size Adjusted BIC (SABIC), and the Bootstrapped Likelihood Ratio Test (BLRT). The model with the lowest AIC, BIC, SABIC, a significant BLRT *p*-value (*p* <.05), and profile sizes greater than 5% of the sample was selected as the best-fitting model [32, 46]. Subsequently, chi-square tests and one-way ANOVA were used to examine differences in characteristics across the latent profiles. Finally, multivariate logistic regression analysis was performed to assess the association between JDCS latent profiles and early retirement intentions. The regression models were adjusted for key covariates, including sex, age, education level, monthly income, household income contribution, occupation, employment type, work experience, shift work, work-family conflict, health problems, and presenteeism. Statistical significance was defined as *p* <.05, and all analyses were conducted using SAS 9.4, while LPA was performed using R (tidyLPA package).

## Results

Participant characteristics and study variables were described using descriptive statistics, and LPA was used to classify job characteristic profiles based on the JDCS model. To determine the optimal number of profile groups, the model was evaluated using the parameters shown in Table 1. We observed the lowest AIC, BIC, SABIC, and significant BLRT *p*-values for the 6-profile solution; however, the 6 profiles were less than 5%, so we chose 5 profiles because the model had lower AIC, BIC, SABIC, and significant BLRT values compared to 2, 3, and 4 profiles.

**Table 1:** Fit indices and model comparisons for the six estimated JDCS profiles

Model	LL (LogLik)	AIC	BIC	SABIC	BLRT(*p*)
1 profile	-136,585.	273,188.	237,261.	2.73e5	< 0.001
2 profiles	-125,454.	250,945.	251,100.	2.51e5	< 0.001
3 profiles	-124,695.	249,477.	249,684.	2.50e5	< 0.001
4 profiles	-124,242.	248,561.	248,880.	2.49e5	< 0.001
**5 profiles**	**-122,930.**	**245,958.**	**246,358.**	**2.46e5**	**< 0.001**
6 profiles	-122,659.	245,435.	245,917.	2.45e5	< 0.001

Notes. AIC, Akaike information criterion; BIC, Bayesian information criterion; BLRT, bootstrapped likelihood ratio test; LL, log-likelihood; SABIC, sample size-adjusted BIC. The bold values indicate that a five-profile model was determined to be optimal

**Fig. 1:**
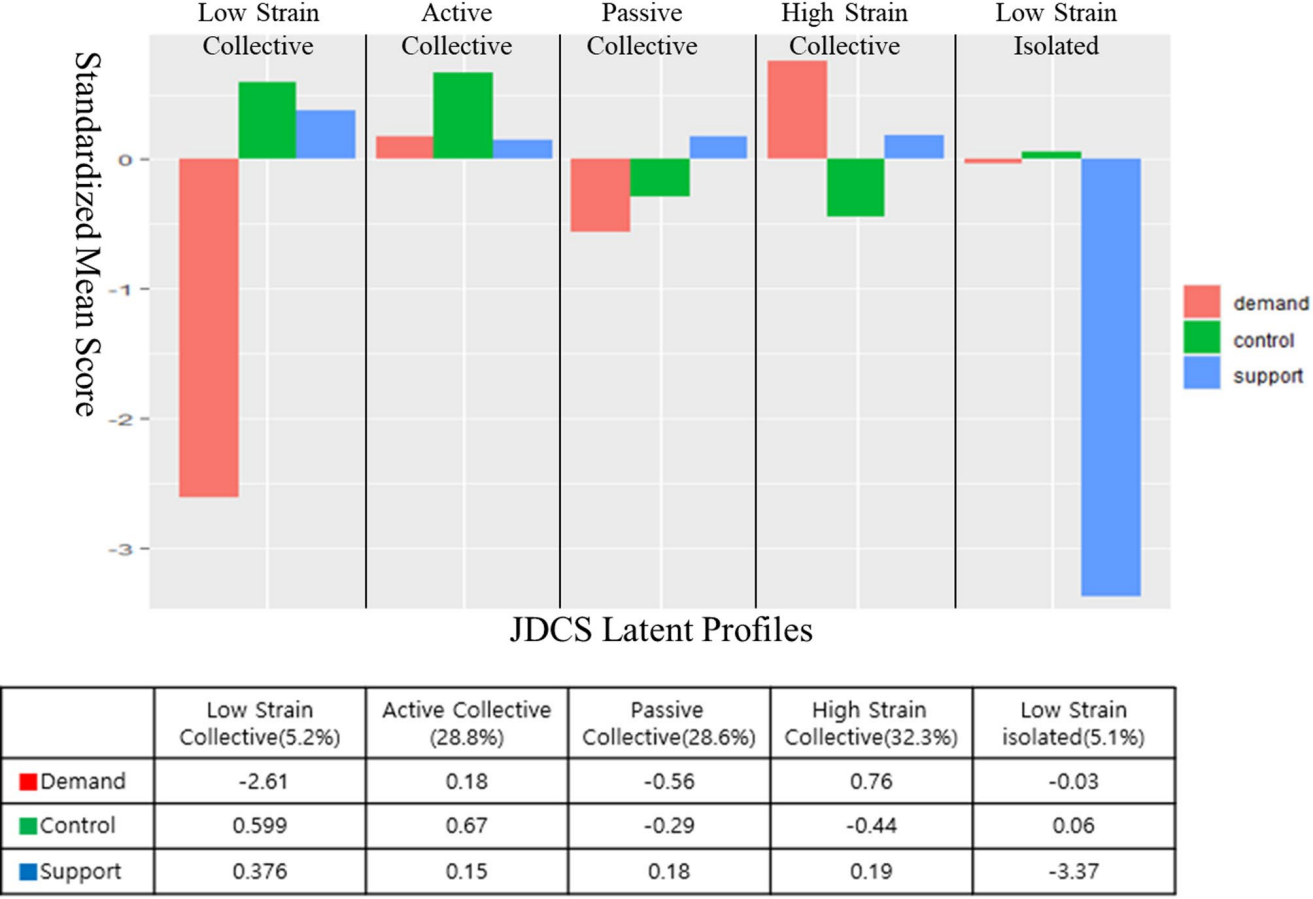
Results from the latent profile analysis (standardized values). Note:Values in the table represent the standardized mean scores for each job characteristic variable (Demand, Control, Support) derived from the LPA based on the JDCS model. Each profile label includes the proportion of the sample (%) classified into that latent group. Positive or negative values indicate the relative position of each variable compared to the sample mean

Figure 1 shows the results for a five-profile solution, where each profile is named in reference to the literature [10]. The first is the ‘Low Strain Collective,’ with low levels of control and support and high levels of demand (*n* = 1,743, 5.52%). The second was the ‘Active Collective,’ with high levels of control, demands, and support (*n* = 8,840, 27.99%). The third, the ‘Passive Collective,’ had low levels of control and demands but high levels of support (*n* = 9,135, 28.92%). The fourth, ‘High Strain Collective,’ had low control but high demand and support (*n* = 10,285, 32.56%). The final group, ‘Low Strain Isolated,’ had high control and low demand and support (*n* = 1,584, 5.01%).

The terms “Low Strain Collective,” “Active Collective,” “Passive Collective,” “High Strain Collective,” and “Low Strain Isolated” are derived from the Demand-Control-Support (JDCS) model, which illustrates how psychological job demands, work control, and work support interact to influence job strain. High control refers to employees having significant autonomy and decision-making authority over their work tasks, whereas low control indicates limited autonomy and decision-making power. High support reflects strong workplace social support from supervisors and coworkers, while low support indicates minimal or no workplace support. High demands refer to jobs with heavy workloads, time pressures, or complex tasks, whereas low demands represent jobs with fewer responsibilities or less intensity. Low Strain Collective represents jobs with low demands, high control, and high support, which typically lead to minimal job strain. Active Collective describes jobs with high demands but also high control and support, where challenges are manageable and may foster motivation and growth. Passive Collective refers to jobs with low demands and low control, which can lead to disengagement or reduced motivation. High Strain Collective represents jobs with high demands, low control, and high support, often resulting in significant stress despite the presence of workplace support. Low Strain Isolated represents jobs with low demands and high control but low support, where job strain is minimized, but employees may experience isolation due to the lack of social support. These classifications demonstrate how varying levels of demand, control, and support influence job strain and workplace well-being.

Table 2 presents the detailed descriptive statistics for the total sample (*N* = 31,587) and across the five latent profile groups identified using the job demand-control-support (JDCS) model: Low Strain Collective, Active Collective, Passive Collective, High Strain Collective, and Low Strain Isolated. Each profile reflects distinct combinations of job demands, job control, and support.

The following variables were measured and are now clarified in the methods section to enhance transparency: Physical Demand was assessed through self-reported frequency of engaging in physically strenuous tasks (e.g., lifting or repetitive motion) on a scale of 0 to 100. Quantitative Demand captured the extent of workload and time pressure (range 1–5), based on validated survey items asking about tight deadlines and fast-paced tasks. Emotional Demand (scale: 1–10) evaluated the degree to which participants dealt with emotionally intense situations or clients. Social Demand referred to the need for interpersonal interaction at work, measured via summated scores of contact frequency with coworkers and customers (range 0–15). Job Control assessed autonomy and discretion over work tasks using a 5-point Likert scale, with higher scores indicating greater control. Job Support represented perceived emotional and practical support from supervisors and colleagues, measured on a composite scale (range 1–10).

The outcome variable, early retirement intention, was a binary item that asked whether the respondent planned to retire before the statutory age, with responses categorized as yes or no.

The sociodemographic and work-related covariates include sex, age, income, education, occupation type, employment type, work experience, and shift work. In addition, three psychosocial or health-related variables were included: Work-Family Conflict, measured on a scale from 4 to 20, assessed the extent to which work interferes with family responsibilities, using four items. Health Problems were based on self-report of chronic conditions lasting more than 6 months. Presenteeism was determined by asking whether the respondent worked while ill during the past 12 months.

Across the total sample, the mean physical demand was 25.36 (SD = 0.02), and the mean quantitative demand was 3.43. Emotional and social demands averaged 6.80 and 11.89, respectively. Mean job control was 4.30, and job support was 8.27.

Gender distribution showed 57.06% of the sample were male, and 42.94% were female. Age-wise, 20.19% were aged 20–29, 25.94% were 30–39, and 53.87% were 40 and older. Educational attainment was high, with 66.47% having a college degree or higher.

Income distribution indicated that 34.02% earned 2,000–3,000 USD per month, followed by 24.08% earning 3,000–4,000 USD, and 19.73% earning over 4,000 USD. Most respondents (63.29%) contributed to household income. Regarding employment, 84.46% held permanent positions, while 11.35% were temporary workers.

Occupational classification included white-collar (27.66%), sales/service (24.72%), skilled manual (18.00%), and elementary occupations (29.62%). In terms of experience, 56.71% had 6–15 years of work history.

Shift work was reported by 10.41% of respondents. Work-family conflict was moderate across the sample (M = 16.51). Health problems were reported by 5.95% of respondents, and presenteeism by 15.89%.

Each latent profile showed distinct characteristics. The Low Strain Collective group (*n* = 1,743) exhibited the lowest physical and quantitative demands but had high job control and the highest job support among all groups. The Active Collective group (*n* = 8,840) demonstrated high demands but also the highest control, suggesting active coping mechanisms. The Passive Collective (*n* = 9,135) had moderate demands and low control but still benefited from higher support. The High Strain Collective (*n* = 10,285) experienced high demands with low control, aligning with classic high-strain job definitions. The Low Strain Isolated (*n* = 1,584) had low demands and moderate control, but extremely low job support, indicating social isolation in the workplace.

For instance, men were overrepresented in the Low Strain Collective (67.13%) and High Strain Collective (57.80%) profiles. The Active Collective had a high proportion of younger workers (25.67% aged 20–29), whereas the Low Strain Isolated group had a majority aged 40+ (64.46%).

Educational attainment was highest in the High Strain Collective (80.55% had college degrees) and lowest in the Low Strain Collective. Monthly incomes above 4,000 USD were most prevalent in the High Strain Collective (28.61%), while lower incomes were more common in the Passive Collective.

Presenteeism was most frequently reported in the Low Strain Collective (30.41%), which might reflect internalized pressure or underrecognized stress. Similarly, health problems were more prevalent in the same group (17.29%).

**Table 2:** Descriptive characteristics of the sample by JDCS profiles (*n* = 31,587)

Variable	Total	JDCS profiles				
		Low Strain Collective	Active Collective	Passive Collective	High Strain Collective	Low-strain Isolated
		*n* = 1,743	*n* = 8,840	*n* = 9,135	*n* = 10,285	*n* = 1,584
		*N*(%) (Bold) or Mean(Std Error of Mean)				
Latent variables						
Physical demand	25.36(0.02)	19.60 (0.14)	25.84(0.02)	24.49 (0.03)	27.01 (0.01)	25.36(0.09)
Quantitative demand	3.43 (0.00)	2.79 (0.03)	3.47 (0.01)	3.17 (0.01)	3.61 (0.01)	3.44 (0.02)
Emotional demand	6.80 (0.01)	6.27 (0.05)	6.83 (0.01)	6.57 (0.01)	7.04 (0.01)	6.75(0.03)
Social demand	11.89 (0.00)	11.72 (0.03)	11.91 (0.01)	11.84(0.01)	11.96 (0.00)	11.88 (0.02)
Job control	4.30 (0.00)	4.67 (0.03)	4.76 (0.01)	4.20 (0.01)	4.10 (0.01)	4.36 (0.03)
Job Support	8.27 (0.02)	9.30 (0.06)	8.95(0.02)	9.03 (0.01)	9.00 (0.01)	2.62 (0.03)
Sex						
Man	**18**,**025 (57.06)**	**1**,**170 (67.13)**	**4**,**387 (49.62)**	**5**,**645 (61.80)**	**5**,**945 (57.80)**	**878 (55.43)**
Woman	**13**,**563 (42.94)**	**573 (32.87)**	**4**,**454 (50.38)**	**3**,**490 (38.20)**	**4**,**340 (42.20)**	**706 (44.57)**
Age						
20–29	**6**,**378 (20.19)**	**402 (23.06)**	**2**,**269 (25.67)**	**1**,**654 (18.11)**	**1**,**806 (17.56)**	**248 (15.66)**
30–39	**8**,**195 (25.94)**	**358 (20.54)**	**2**,**195(24.83)**	**2**,**345 (25.67)**	**2**,**981 (28.98)**	**315 (19.89)**
> 40	**17**,**015 (53.87)**	**983 (56.40)**	**4**,**376 (49.50)**	**5**,**136 (56.22)**	**5**,**499 (53.46)**	**1**,**021 (64.46)**
Monthly income (USD )						
< 2,000	**5**,**287 (22.17)**	**386 (27.57)**	**2**,**111 (31.42)**	**1**,**358 (19.42)**	**1**,**105 (14.36)**	**327 (31.32)**
2,000–3,000	**8**,**114 (34.02)**	**429(30.64)**	**2**,**430 (36.17)**	**2**,**568 (36.73)**	**2**,**396 (31.15)**	**290 (27.78)**
3,000–4,000	**5**,**742 (24.08)**	**399(28.50)**	**1**,**257 (18.71)**	**1**,**851 (26.48)**	**1**,**991 (25.88)**	**244(23.37)**
> 4,000	**4**,**706 (19.73)**	**186 (13.29)**	**921(13.71)**	**1**,**214 (17.37)**	**2**,**201 (28.61)**	**183 (17.53)**
Educational level						
Elementary school or less	**103(0.33)**	**17(0.98)**	**37(0.42)**	**25(0.27)**	**9 (0.09)**	**15(0.96)**
Middle school	**490 (1.55)**	**54 (3.11)**	**190 (2.15)**	**160(1.75)**	**51(0.50)**	**35 (2.24)**
High school	**9**,**982 (31.65)**	**952 (54.81)**	**3**,**236 (36.61)**	**3**,**318 (36.37)**	**1**,**938 (18.86)**	**538 (34.40)**
College degree or higher	**20**,**961 (66.47)**	**714 (41.11)**	**5**,**375 (60.82)**	**5**,**619 (61.60)**	**8**,**277 (80.55)**	**976 (62.40)**
Household income contribution						
Yes	**19**,**923 (63.29)**	**1**,**192 (68.70)**	**4**,**866 (55.19)**	**6**,**215 (68.24)**	**6**,**669 (64.95)**	**981 (63.21)**
No	**11**,**557 (36.71)**	**543 (31.30)**	**3**,**951 (44.81)**	**2**,**893 (31.76)**	**3**,**599 (35.05)**	**571 (36.79)**
Type of occupation						
White color	**8**,**737 (27.66)**	**182(10.44)**	**2**,**108(23.84)**	**2**,**271(24.86)**	**3**,**684 (35.82)**	**492 (31.08)**
Sales/service	**7**,**807 (24.72)**	**99 (5.68)**	**2**,**027(22.93)**	**1**,**531 (16.76)**	**3**,**861 (37.54)**	**289 (18.26)**
Skilled manual	**5**,**688 (18.00)**	**291(16.70)**	**1**,**942 (21.97)**	**1**,**701 (18.62)**	**1**,**414 (13.75)**	**340 (21.48)**
Elementary	**9**,**355 (29.62)**	**1**,**171 (67.18)**	**2**,**764 (31.26)**	**3**,**632 (39.76)**	**1**,**326 (12.89)**	**462 (29.19)**
Employment type						
Permanent	**26**,**680 (84.46)**	**1**,**275 (73.15)**	**7**,**046 (79.71)**	**7**,**758 (84.93)**	**9**,**436 (91.75)**	**1**,**165 (73.55)**
Temporary	**3**,**585 (11.35)**	**240(13.77)**	**1**,**340 (15.16)**	**989 (10.83)**	**727 (7.07)**	**289 (18.24)**
Others	**1**,**322 (4.19)**	**228 (13.08)**	**454 (5.14)**	**388 (4.25)**	**122 (1.19)**	**130 (8.21)**
Work experience						
0–5 years	**7**,**366 (23.53)**	**579(33.74)**	**2**,**605 (29.67)**	**1**,**991 (21.93)**	**1**,**795 (17.55)**	**396 (26.42)**
6–15 years	**17**,**751 (56.71)**	**834(48.60)**	**4**,**893 (55.72)**	**5**,**196 (57.24)**	**6**,**030(58.960**	**798 (53.24)**
>= 16 years	**6**,**184 (19.76)**	**303 (17.66)**	**1**,**283 (14.61)**	**1**,**890 (20.82)**	**2**,**403 (23.49)**	**305(20.35)**
Shift work						
Yes	**3**,**279 (10.41)**	**400 (23.05)**	**1**,**083 (12.28)**	**1**,**169 (12.83)**	**515 (5.02)**	**112 (7.12)**
No	**28**,**210 (89.59)**	**1**,**335 (76.95)**	**7**,**734 (87.72)**	**7**,**940 (87.17)**	**9**,**740 (94.98)**	**1**,**461 (92.88)**
Work-family-conflict (4–20 points)	16.51 (0.02)	15.26 (0.17)	16.89(0.06)	16.30 (0.05)	16.86(0.05)	16.26 (0.13)
Health problem						
Yes	**1**,**871(5.95)**	**299 (17.29)**	**544 (6.17)**	**515 (5.65)**	**446 (4.34)**	**67 (4.36)**
No	**295**,**85(94.05)**	**1**,**430 (82.71)**	**8**,**267 (93.83)**	**8**,**601 (94.35)**	**9**,**819(95.66)**	**1**,**468 (95.64)**
Presenteeism						
Yes	**3**,**473 (15.89)**	**419(30.41)**	**937 (15.52)**	**1**,**165(17.45)**	**830 (12.32)**	**122(11.72)**
No	**18**,**396 (84.12)**	**959 (69.59)**	**5**,**100 (84.48)**	**5**,**512(82.55)**	**5**,**906 (87.68)**	**919 (88.28)**

Table 3 presents the distribution of early retirement intentions according to various participant characteristics, offering a detailed comparison between those who intend to retire early and those who do not. The table is structured to display the frequency and percentage (in bold) or mean and standard error of the mean across JDCS latent profile groups, job demand variables, and sociodemographic and occupational factors. Significant differences were observed across most variables, as indicated by chi-square or t-tests.

Regarding JDCS profiles, individuals categorized within the Active Collective group exhibited the highest rate of early retirement intention (12.22%), followed by the High Strain Collective (11.83%) and Passive Collective (8.46%). In contrast, the Low Strain Isolated and Low Strain Collective groups reported lower rates, at 6.97% and 7.96%, respectively. This indicates that higher levels of job strain and emotional demand, coupled with collective work environments, are associated with elevated early retirement intentions.

In terms of latent job-related variables, individuals intending to retire early reported significantly higher physical (25.84 vs. 25.43, *p* <.0001) and quantitative demands (3.43 vs. 3.39, *p* =.003), but slightly lower emotional demands (6.73 vs. 6.80, *p* =.001). They also reported higher levels of job control (4.41 vs. 4.35, *p* <.0001) and support (8.83 vs. 8.68, *p* <.0001), suggesting a nuanced interaction between psychological demands and perceived workplace support in retirement decision-making.

Sociodemographic characteristics also influenced early retirement intentions. Female participants were more likely than males to consider early retirement (15.66% vs. 7.17%, *p* <.0001). Younger workers, especially those aged 20–29, had the highest early retirement intentions (19.35%), followed by those in their 30s (61.52%), while only 6.30% of those aged over 40 expressed such intentions. This suggests age is inversely associated with early retirement intention, potentially reflecting younger workers’ expectations or dissatisfaction.

Economic variables were also predictive. Participants with monthly incomes below 2,000 USD were most likely to intend early retirement (15.16%), while the rate decreased as income rose, with only 6.59% of those earning above 4,000 USD expressing early retirement intentions. Similarly, individuals who did not contribute to household income were significantly more likely to intend early retirement (16.58%) compared to those who did (7.53%). Education was also a factor, with the highest proportion of early retirement intention found among those with college or higher education (11.99%), suggesting that higher education may foster expectations of career flexibility or exit strategies.

Occupational characteristics revealed that temporary workers had the highest rate of early retirement intention (16.20%) compared to permanent workers (10.16%). Among occupations, white-collar workers showed the highest retirement intention (13.11%), while those in elementary jobs had the lowest (6.72%). Work experience had a strong inverse relationship with early retirement intentions; those with 0–5 years of experience reported the highest intention (14.62%), followed by those with 6–15 years (10.85%), and only 6.24% of those with 16 years or more.

Although shift work and work-family conflict were not significantly associated with early retirement intentions (*p* =.669 and *p* =.721, respectively), health-related factors were. Participants without reported health problems were more likely to intend early retirement (10.97%) compared to those with health issues (8.05%, *p* =.011), which may suggest that better health enables proactive planning for retirement. Presenteeism showed a strong association with early retirement intentions; 12.90% of those reporting presenteeism considered early retirement, compared to 9.57% of those without it (*p* <.0001).

In summary, Table 3 illustrates that early retirement intention is influenced by a complex interplay of psychological work profiles, sociodemographic characteristics, and employment conditions. Workers experiencing higher job strain, lower income, and insecure employment—especially among younger, female, and highly educated groups—are more likely to express early retirement intentions. These findings suggest that targeted workplace interventions addressing job demands, employment stability, and employee well-being could mitigate early workforce exits.

**Table 3:** Distribution of early retirement intentions according to participant characteristics

Variable	Early Retirement Intentions			
	No	Yes	χ^2^ or t	*p*
	*N*(%) (Bold) or Mean(Std Error of Mean)			
Profiles				
Low Strain Collective	**1**,**550 (92.04)**	**134(7.96)**	33.09	< 0.0001
Active Collective	**7**,**553 (87.78)**	**1**,**051 (12.22)**		
Passive Collective	**8**,**136(90.54)**	**850 (8.46)**		
High Strain Collective	**8**,**985 (88.17)**	**1**,**206 (11.83)**		
Low Strain Isolated	**1**,**415 (93.03)**	**106 (6.97)**		
Latent variables				
Physical demand	25.43(0.02)	25.84(0.06)	6.63	< 0.0001
Quantitative demand	3.39 (0.01)	3.43 (0.01)	2.98	0.003
Emotional demand	6.80 (0.01)	6.73 (0.02)	-3.23	0.001
Social demand	11.90 (0.00)	11.88 (0.01)	-1.37	0.170
Job control	4.35 (0.01)	4.41 (0.02)	3.69	< 0.0001
Job Support	8.68 (0.02)	8.83 (0.04)	3.52	< 0.0001
Sex			207.92	< 0.0001
Man	**16**,**448 (92.83)**	**1**,**270 (7.17)**		
Woman	**11**,**190 (84.34)**	**2**,**077 (15.66)**		
Age			325.86	< 0.0001
20–29	**4**,**915 (80.65)**	**1**,**179 (19.35)**		
30–39	**6**,**949 (38.48)**	**11**,**109 (61.52)**		
> 40	**15**,**775 (93.70)**	**1**,**060 (6.30)**		
Monthly income(USD)			91.43	< 0.0001
< 2,000	**4**,**320 (84.84)**	**772 (15.16)**		
2,000–3,000	**6**,**980 (87.06)**	**1**,**037(12.94)**		
3,000–4,000	**5**,**187 (90.87)**	**521(9.13)**		
> 4,000	**4**,**364 (93.41)**	**308 (6.59)**		
Educational level			70.42	< 0.0001
Elementary school or less	**102 (99.03)**	**1(0.97)**		
Middle school	**471 (96.71)**	**16 (3.29)**		
High school	**8**,**852 (91.19)**	**855 (8.81)**		
College degree or higher	**18**,**174 (88.01)**	**2**,**475 (11.99)**		
Household income contribution			228.92	< 0.0001
Yes	**18**,**199 (92.47)**	**1**,**482 (7.53)**		
No	**9**,**362 (83.42)**	**1**,**861 (16.58)**		
Type of occupation			96.41	< 0.0001
White color	**7**,**500 (86.89)**	**1**,**132 (13.11)**		
Sales/service	**6**,**875 (89.30)**	**824 (10.70)**		
Skilled manual	**4**,**726 (85.90)**	**776 (14.10)**		
Elementary	**8**,**538 (93.28)**	**615(6.72)**		
Employment type			92.41	< 0.0001
Permanent	**23**,**660 (89.84)**	**2**,**675 (10.16)**		
Temporary	**2**,**834 (83.80)**	**548 (16.20)**		
Others	**1**,**145(90.23)**	**124(9.77)**		
Work experience			83.73	< 0.0001
0–5 years	**6**,**019 (85.38)**	**1**,**031 (14.62)**		
6–15 years	**15**,**619 (89.15)**	**1**,**900 (10.85)**		
>=16 years	**5**,**767 (93.76)**	**384 (6.24)**		
Shift work			0.18	0.669
Yes	**2**,**809 (88.84)**	**353 (11.16)**		
No	**24**,**742 (89.23)**	**2986 (10.77)**		
Work-family-conflict			0.36	0.721
Mean (4 to 20 points)	16.58 (0.03)	16.62 (0.09)		
Health problem			6.40	0.011
Yes	**1**,**691 (91.95)**	**148 (8.05)**		
No	**25**,**861 (89.03)**	**3**,**185(10.97)**		
Presenteeism			15.54	< 0.0001
Yes	**2**,**971 (87.10)**	**440 (12.90)**		
No	**16**,**341 (90.43)**	**1**,**729 (9.57)**		

Table 4 presents the results of a single multivariate logistic regression model that examined the association between latent job profiles and early retirement intentions, while simultaneously adjusting for a range of sociodemographic and work-related covariates. This single model approach was selected for parsimony and to provide a comprehensive view of the adjusted associations in one unified analysis. The statistical methods section has been updated accordingly to clarify that a fully adjusted model was estimated to control for potential confounders.

Results showed that compared to the Low Strain Collective group (reference category), individuals in the Active Collective (OR = 1.65, 95% CI: 1.10–2.48), Passive Collective (OR = 1.72, 95% CI: 1.15–2.60), and High Strain Collective groups (OR = 1.66, 95% CI: 1.10–2.49) had significantly higher odds of early retirement intentions. The Low Strain Isolated group did not show a significant difference from the reference category. These findings suggest that both high job demands and varying levels of support and control may influence retirement decision-making.

Among covariates, gender was a significant predictor: women had 1.97 times higher odds of intending to retire early than men (95% CI: 1.60–2.43). Age also played a key role; individuals aged 40 or older were significantly less likely to intend early retirement compared to those in their 20s (OR = 0.40, 95% CI: 0.32–0.50). Education had a strong effect: individuals with a high school education were 4.31 times more likely, and those with a college degree or higher were 5.89 times more likely, to express early retirement intentions compared to those with elementary education or less. Lack of contribution to household income (OR = 1.29, 95% CI: 1.05–1.58), being employed as a temporary worker (OR = 1.65, 95% CI: 1.29–2.12), and experiencing presenteeism (OR = 1.40, 95% CI: 1.14–1.73) were also associated with higher early retirement intentions. However, individuals in sales/service jobs were less likely to express early retirement intentions compared to those in white-collar occupations (OR = 0.80, 95% CI: 0.65–0.98).

**Table 4:** Odds ratios of the JDCS profiles and early retirement intentions

Variable	Early Retirement Intentions	
	Odds ratio	95% CI
Low Strain Collective	1.00	
Active Collective	1.65	1.10–2.48
Passive Collective	1.72	1.15–2.60
High Strain Collective	1.66	1.10–2.49
Low Strain Isolated	1.21	0.70–2.10
Sex		
Man	1.00	
Woman	1.97	1.60–2.43
Age		
20–29	1.00	
30–39	0.82	0.64–1.06
> 40	0.40	0.32–0.50
Monthly income (USD)		
< 2,000	1.00	
2,000–3,000	1.13	0.91–1.41
3,000–4,000	1.18	0.88–1.59
> 4,000	0.88	0.63–1.23
Educational level		
Elementary school or less	1.00	
Middle school	1.45	0.28–7.62
High school	4.31	1.02–18.34
College degree or higher	5.89	1.39–25.05
Household income contribution		
Yes	1.00	
No	1.29	1.05–1.58
Type of occupation		
White color	1.00	
Sales/service	0.80	0.65–0.98
Skilled manual	0.93	0.73–1.19
Elementary	0.80	0.60–1.07
Employment type		
Permanent	1.00	
Temporary	1.65	1.29–2.12
Others	1.43	0.88–2.34
Work experience		
0–5 years	1.00	
6–15 years	0.92	0.72–1.17
>=16 years	0.95	0.66–1.35
Shift work		
No	1.00	
Yes	1.20	0.93–1.55
Work-family-conflict	1.00	0.98–1.03
Health problem		
Yes	1.00	
No	1.36	0.94–1.96
Presenteeism		
No	1.00	
Yes	1.40	1.14–1.73
Nagelkerke *R²* = 0.32		

Note: Results are from a fully adjusted multivariate logistic regression model. Reference groups are indicated. CI = Confidence Interval

## Discussion and conclusion

This study explored the relationship between job demands, job control, and job support and their association with early retirement intentions among Korean wage earners. By applying LPA based on the JDCS model, we identified five distinct worker profiles: Low Strain Collective, Active Collective, Passive Collective, High Strain Collective, and Low Strain Isolated. Our findings revealed that workers in the Active Collective, Passive Collective, and High Strain Collective groups were significantly more likely to express early retirement intentions compared to those in the Low Strain Collective group, which was characterized by low demands and high control and support. This highlights the substantial role of the psychosocial work environment in shaping retirement preferences.

A growing body of research supports the idea that job resources—particularly job control and social support—serve as protective factors against premature labor market exits. In our study, individuals in profiles with greater job control and support, such as the Low Strain Collective group, had the lowest early retirement intentions. This is consistent with findings from P Browne, E Carr, M Fleischmann, B Xue and SA Stansfeld [16], who, in a systematic review, concluded that both social support and job control are associated with delayed retirement. Although methodological heterogeneity in measuring social support made it difficult to disaggregate its effects in that review, supervisor and coworker support were particularly influential. Similarly, K Nilsson [29] reported that Swedish women who lacked perceived support from supervisors and colleagues were less likely to remain in employment until the statutory retirement age. These insights underscore the role of organizational support structures in promoting longer working lives.

Workplace culture that encourages equality, open communication, and mutual respect also appears to reduce retirement intentions. S Neupane, S Kyrönlahti, H Kosonen, KC Prakash, A Siukola, K Lumme-Sandt, P Nikander and CH Nygård [15] demonstrated that perceptions of fairness and equality in the workplace community—such as being valued and respected—correlated with lower retirement intentions. In the context of our study, this suggests that supportive environments not only buffer the effects of high job demands but also foster a sense of purpose and engagement that can encourage prolonged labor force participation.

Supervisors play a particularly important role in this dynamic. As noted by A Min and HC Hong [31], when supervisors actively regulate workload distribution and provide employees with appropriate levels of decision-making authority, they reduce employee stress and presenteeism, which are known risk factors for early exit from the workforce. Other studies confirm that supervisor support significantly improves job satisfaction and mitigates burnout [18, 47]. A strong support system at work, therefore, can enhance employee well-being and encourage continued employment.

Job control also emerged as a key predictor of retirement intention. Workers with greater autonomy over how and when tasks are performed reported lower early retirement intentions. This aligns with the findings of M ten Have, S van Dorsselaer and R de Graaf [48], who noted that low decision authority was a significant predictor of early retirement preferences. The interplay between job control and support is especially noteworthy. E Carr, G Hagger-Johnson, J Head, N Shelton, M Stafford, S Stansfeld and P Zaninotto [34] suggest that social support and recognition often co-occur with job autonomy, which together reduce job stress and delay retirement. H Halvari and AH Olafsen [17] also found that workers in environments characterized by high autonomy and intrinsic motivation experienced less fatigue and job stress, further supporting our findings.

Regarding job demands, the findings of this study offer a nuanced perspective. While P Browne, E Carr, M Fleischmann, B Xue and SA Stansfeld [16] found limited evidence linking job demands to early retirement intentions, subsequent research has emphasized the detrimental impact of excessive physical and psychological demands. For instance, K Nilsson [29] and S Neupane, S Kyrönlahti, H Kosonen, KC Prakash, A Siukola, K Lumme-Sandt, P Nikander and CH Nygård [15] found that physically demanding work, awkward postures, and a fast work pace reduced the likelihood of working to the official retirement age. Our study found similar results, particularly among members of the High Strain Collective group, who experienced high physical and quantitative demands and expressed higher retirement intentions. Moreover, Alkaabi and FA Alkaabi and PK Maghelal [20] demonstrated that exposure to a range of occupational hazards—vibrations, noise, and chemical exposure—increased early retirement intentions among Abu Dhabi police officers. These findings are echoed in European research showing that prolonged fatigue and physically intense work are key drivers of early retirement [49, 50].

Increased job demands, particularly those involving exposure to hazardous materials, rapid work pace, emotional labor, and experiences of workplace violence or bullying, have been consistently associated with negative health outcomes for workers. Such stressors contribute to both psychological strain—manifesting as stress, anxiety, and burnout—and physiological conditions, including fatigue and sleep disturbances [22, 51, 52]. These consequences are especially pronounced among older workers, for whom prolonged physical labor and chronic fatigue pose significant challenges to sustained workforce participation. Prior research highlights that these cumulative stressors are strong predictors of early retirement intentions [49, 50]. Our findings support this perspective, showing that higher physical and quantitative job demands were significantly associated with increased intentions to exit the workforce prematurely.

Importantly, workers in better physical and mental health or in less demanding roles are more likely to extend their participation in the labor force. According to A Meng, E Sundstrup and LL Andersen [19], favorable health status and manageable workloads are key determinants in the decision to delay retirement. Conversely, demanding work environments may accelerate withdrawal from employment, particularly when combined with insufficient organizational support or autonomy. This dynamic aligns with our study’s results: job demands alone do not predict early retirement in isolation but interact significantly with perceived support and control.

Beyond the psychosocial factors, several individual and work-related characteristics were also significantly associated with early retirement intentions. For example, while we did not observe a direct association between absolute income levels and retirement intentions, a significant pattern emerged with respect to household income contribution. Participants who reported not contributing to their household income demonstrated a stronger inclination toward early retirement. This observation aligns with findings from KG Reeuwijk, A de Wind, MJ Westerman, JF Ybema, AJ van der Beek and GA Geuskens [11] and R Sewdas, A de Wind, LGL van der Zwaan, WE van der Borg, R Steenbeek, AJ van der Beek and CRL Boot [53], who emphasized the social and relational aspects of retirement decisions. These studies suggest that retirement timing is often influenced by spousal retirement plans, shared leisure goals, and family dynamics. Particularly, some older individuals may prefer to retire when their spouse does, or conversely, delay retirement to avoid social isolation if the spouse remains employed.

Employment status also played a central role in shaping retirement preferences. Consistent with previous findings, workers in precarious employment situations—such as temporary or irregular contracts—were more likely to report early retirement intentions. This is consistent with research by YJ Yang and J Lee [54], who found that precarious workers are at elevated risk for stress-related disorders, including depression, due to job insecurity. In contrast, permanent employees reported lower early retirement intentions, likely due to the job security and organizational attachment that full-time employment offers [55]. Secure employment not only provides economic stability but also reinforces psychological contract fulfillment, enhancing commitment and reducing turnover intention [56].

Presenteeism—defined as working while unwell—also emerged as a significant predictor. Participants who engaged in presenteeism were more likely to express a desire for early retirement. This finding is supported by YS Cho, JB Park, KJ Lee, KB Min and CI Baek [57], who noted that presenteeism erodes physical and mental well-being and negatively affects productivity. Moreover, FA Alkaabi and PK Maghelal [20] argue that chronic presenteeism may contribute to disengagement and reduced occupational satisfaction, ultimately motivating employees to consider early exit from the labor force. Therefore, addressing presenteeism may be critical not only for health promotion but also for workforce retention strategies.

The JDCS model offers a robust framework for understanding how workplace conditions influence early retirement. This model posits that high demands, low control, and insufficient support create high-strain environments, which, over time, lead to negative outcomes such as burnout and disengagement. Our study empirically supports this theoretical framework: participants in the Low Strain Collective group—characterized by low demands and high control and support—had the lowest early retirement intentions, while those in High Strain or Passive profiles were more likely to plan early retirement.

Organizational culture significantly shapes the levels of demands, control, and support workers perceive. A culture emphasizing high productivity without providing corresponding autonomy or support increases job strain, which can fuel early retirement decisions. Conversely, cultures promoting teamwork, transparency, participatory decision-making, and supportive leadership reduce stress and enhance workers’ willingness to remain employed. Evidence from JP Edwards and PL Solomon [18] and A Orgambídez, H Almeida and Y Borrego [47] confirms that environments with high supervisor and coworker support are associated with delayed retirement intentions and increased job satisfaction.

Moreover, workplace cultures that prioritize fairness, recognition, and worker empowerment can buffer the adverse effects of high demands by enhancing job resources. For instance, when employees feel fairly treated and valued, they are more likely to remain committed to their roles even under demanding conditions. Thus, a focus on organizational culture interventions can be a strategic avenue to extend the working lives of aging employees, particularly in countries like South Korea, where workforce aging poses significant policy challenges.

Based on our findings, several recommendations emerge. First, organizations should actively monitor and moderate job demands to prevent overexertion and protect employees’ health. This includes minimizing unnecessary physical tasks, managing emotional labor, and ensuring appropriate pacing of work. Occupational health policies should integrate strategies to assess and reduce exposure to psychosocial risks, especially for aging workers. Second, enhancing worker autonomy is essential. Employees should be given greater control over how they execute tasks, as increased decision-making authority is linked to lower stress and longer employment. Flexibility in task execution or scheduling can serve as a buffer against high demands. Third, organizations must invest in developing supportive work environments. This includes fostering open communication, encouraging collaboration, and training supervisors to provide meaningful support. Finally, policy-level interventions are warranted to create incentives for employers to retain older workers through ergonomic accommodations, phased retirement options, and lifelong learning initiatives.

In summary, early retirement intentions are not shaped by singular factors but emerge from a complex interplay between individual characteristics and workplace conditions. By strategically improving job design, enhancing workplace support, and fostering inclusive organizational cultures, it is possible to mitigate early retirement risks and retain experienced workers in the labor market.

There are three limitations to this study. The first is that it is a cross-sectional study, so it is difficult to establish a causal relationship between job demands, control, support, and early retirement intentions. A longitudinal study, such as a cohort study, could complement this study. Second, the study did not distinguish between specific occupational groups or age groups, so future studies may benefit from examining early retirement intentions by occupational group or age group. Third, the study was conducted during the COVID-19 pandemic, so the rapidly changing work environment may have been different from today, and the use of secondary data analysis may have excluded confounding variables. Finally, this study is limited by the use of Korea-specific retirement age standards, which may not align with global norms and could influence the generalizability of the findings. Future research should apply international age standards and adopt statistical techniques designed for rare events to provide broader and more robust insights.

Despite these limitations, this study has strengths in that it uses questionnaires and data from Korean workers to identify the impact of subgroup characteristics, weighted by psychosocial factors, on early retirement intentions.

## Data Availability

The data analyzed in this study can be downloaded from the following sites https://oshri.kosha.or.kr/oshri/researchField/downWorkingEnvironmentSurvey.do.
